# Low-Intensity Pulsed Ultrasound Stimulation Facilitates Osteogenic Differentiation of Human Periodontal Ligament Cells

**DOI:** 10.1371/journal.pone.0095168

**Published:** 2014-04-17

**Authors:** Bo Hu, Yuanyuan Zhang, Jie Zhou, Jing Li, Feng Deng, Zhibiao Wang, Jinlin Song

**Affiliations:** 1 Chongqing key Laboratory for Oral Diseases and Biomedical Sciences, Chongqing, China; 2 College of Stomatology, Chongqing Medical University, Chongqing, China; 3 Wake Forest Institute for Regenerative Medicine, Wake Forest School of Medicine, Winston-Salem, North Carolina, United States of America; 4 College of Biomedical Engineering, Chongqing Medical University, Chongqing, China; Boston University Goldman School of Dental Medicine, United States of America

## Abstract

Human periodontal ligament cells (hPDLCs) possess stem cell properties, which play a key role in periodontal regeneration. Physical stimulation at appropriate intensities such as low-intensity pulsed ultrasound (LIPUS) enhances cell proliferation and osteogenic differentiation of mesechymal stem cells. However, the impacts of LIPUS on osteogenic differentiation of hPDLCs *in vitro* and its molecular mechanism are unknown. This study was undertaken to investigate the effects of LIPUS on osteogenic differentiation of hPDLCs. HPDLCs were isolated from premolars of adolescents for orthodontic reasons, and exposed to LIPUS at different intensities to determine an optimal LIPUS treatment dosage. Dynamic changes of alkaline phosphatase (ALP) activities in the cultured cells and supernatants, and osteocalcin production in the supernatants after treatment were analyzed. Runx2 and integrin β1 mRNA levels were assessed by reverse transcription polymerase chain reaction analysis after LIPUS stimulation. Blocking antibody against integrinβ1 was used to assess the effects of integrinβ1 inhibitor on LIPUS-induced ALP activity, osteocalcin production as well as calcium deposition. Our data showed that LIPUS at the intensity of 90 mW/cm^2^ with 20 min/day was more effective. The ALP activities in lysates and supernatants of LIPUS-treated cells started to increase at days 3 and 7, respectively, and peaked at day 11. LIPUS treatment significantly augmented the production of osteocalcin after day 5. LIPUS caused a significant increase in the mRNA expression of Runx2 and integrin β1, while a significant decline when the integrinβ1 inhibitor was used. Moreover, ALP activity, osteocalcin production as well as calcium nodules of cells treated with both daily LIPUS stimulation and integrinβ1 antibody were less than those in the LIPUS-treated group. In conclusion, LIPUS promotes osteogenic differentiation of hPDLCs, which is associated with upregulation of Runx2 and integrin β1, which may thus provide therapeutic benefits in periodontal tissue regeneration.

## Introduction

Congenital malformations, trauma or periodontal disease and other factors often lead to different degrees of alveolar bone defects, injuring to tooth supporting tissues, causing damages to periodontal attachment loss, eventually leading to loss of tooth [1]. Currently, several main treatment modalities such as periodontal bone grafting, guided tissue regeneration and guided bone regeneration have been developed for periodontal tissue repair [1], [2]. However, the clinical outcomes of these approaches vary tremendously among individual patients [3], [4]. During wound healing, regeneration of periodontal tissue is derived from ancestral cells in periodontal ligament and bone. Periodontal ligament cells (PDLCs), derived from periodontal ligament, are a heterogeneous cell population [5], including mesenchymal stem cells with self-renewal and multipotent differentiation potential [6], [7]. Like bone marrow stromal cells, PDLCs have the ability to give rise to mesoderm cell lineages, such as alveolar bone, cementum, and periodontal ligament for periodontal tissue regeneration [4], [8]. The major goal of periodontal therapy is to prevent further attachment loss and predictably restore the periodontal supporting structures [9]. New bone formation is critical for maintaining the structural stability and physiological function of the dentition [10], so promoting osteogenic differentiation of PDLCs during wound healing and regeneration is significative. Most PDLCs in periodontal lesion tissues, however, require the inflammatory-free microenvironments and biological activity for periodontal wound healing. The addition of exogenous growth factors has been shown to improve osteogenic differentiation of PDLCs [11], but large-scale utilization of growth factors is clinically impracticable due to safety concern [12]. Therefore, it is of significance to develop new approaches to improve the osteogenic potential of PDLCs.

Appropriate intensities of physical stimulation, enhance cellular metabolism and phenotypic adaptation. The physical stimulation includes physical excise [13], low current electric stimulation [14], and low-intensity pulsed ultrasound (LIPUS) [15]. LIPUS with intensities of 30–100 mW/cm^2^, is a form of mechanical energy that is transmitted through and into living tissues as an acoustic pressure wave, resulting in biochemical events at the cellular level [16]. LIPUS, as a bio-physical therapy, is a safe and effective approach approved for the treatment of fresh bone fractures [17] by the Food and Drug Administration (FDA) in 1994. The therapy has offered several advantages in periodontal regeneration, including noninvasiveness, minimal adverse tissue reactions, and easy to handle [18]. LIPUS has been reported to accelerate healing of the resorption by reparative cementum during experimental tooth movement in orthodontic patients [19]. In addition, this treatment has been shown to stimulate periodontal wound healing via enhancement of new bone and cementum formation [20]. Furthermore, several *in vitro* studies have confirmed the upregulation of a variety of genes related to mineral metabolism [16], cementoblastic differentiation [21], and fibroblast differentiation [22] by LIPUS. Our recent studies demonstrated the beneficial effects of LIPUS in repairing the alveolar bone defect [23] and periodontal fenestration defect [24], and promoting proliferation of hPDLCs *in vitro* [25]. Despite extensive studies on the pronounced effects of LIPUS stimulation, few reports have shown the effects of LIPUS on osteogenic differentiation of PDLCs and its molecular mechanism. In the present study, we conducted an *in vitro* study to investigate the effects of LIPUS on osteogenic differentiation potential of hPDLCs and preliminarily analysed the underlying mechanisms, which is potentially used for periodontal tissue engineering.

## Materials and Methods

### Ethics Statement

The study was approved by the ethics committee of the Affiliated Hospital of Stomatology, Chongqing Medical University, Chongqing, China. And written informed consent was obtained from all the guardians of 10 healthy adolescents, who would take the orthodontic extraction treatment.

### Isolation, culture and identification of hPDLCs

The extracted premolars (n =  60) of the adolescents were collected and PDLCs were isolated from the premolar tissue samples as described previously [26]. Briefly, the periodontal ligament tissue was taken from the mid-third portion of the premolar root and cut into ∼1-mm^3^ pieces. The tissue pieces were then cultured in Dulbecco's modified Eagle's medium (DMEM; Hyclone, Logan, UT, USA) with 10% fetal bovine serum (FBS; Hyclone), 100 µg/mL streptomycin, and 100 U/mL penicillin at 37°C in an incubator with 95% air and 5% CO_2_. HPDLCs outgrown from the tissue explants were subcultured when they reached ∼60% confluence. Cells at passage 5 were used for the following experiments.

The cells were characterized morphologically under an inverted microscope (Olympus, Japan). Cells on tissue culture-treated coverslips were examined immunohistochemically using anti-keratin antibody and anti-vimentin antibody [27].

### LIPUS exposure protocol

**Figure 1:**
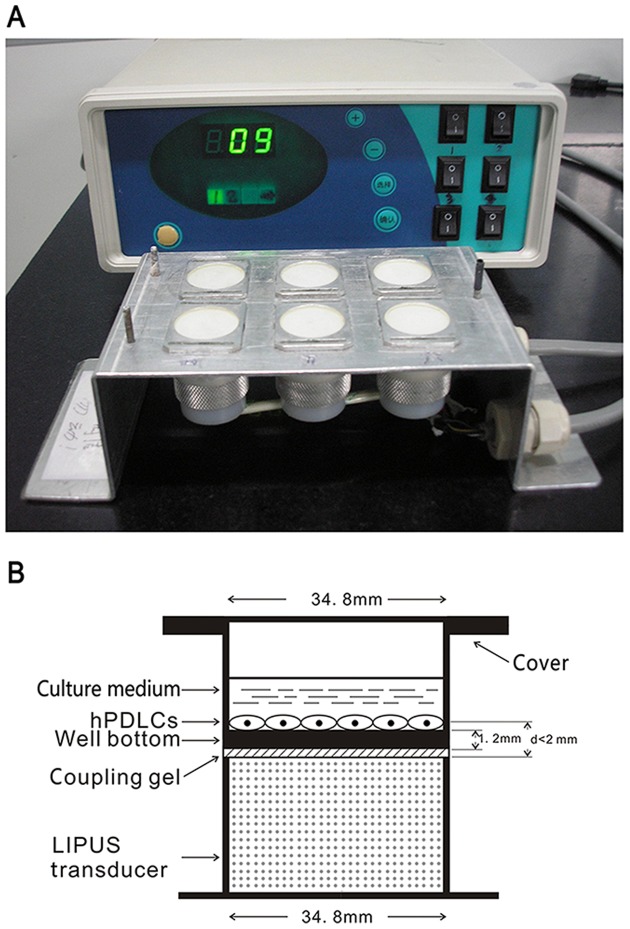
Schematic illustration of ultrasound device and procedure. (A) Schematic view of the LIPUS exposure device used in this study, which consists of an array of 6 transducers. (B) Schematic illustration showing that a culture plate is placed on the ultrasound transducer array with a thin layer of ultrasonic coupling gel, and the distance between the transducer and cells was less than 2 mm.

The LIPUS exposure device manufactured by National Engineering Research Center of Ultrasound Medicine (Chongqing Medical University, Chongqing, China) consists of an array of 6 transducers (each with a diameter of 34.8 mm), which are specifically designed for use in a 6-well culture plate (Fig. 1A). Two days before ultrasound treatment, hPDLCs were seeded onto 6-well culture plates at a density of 5×10^4^ cells per well (diameter = 34.8 mm, surface area = 9.5 cm^2^, and thickness of the well bottom = 1.2 mm). After incubation, the culture plates were placed on the ultrasound transducer array with a thin layer of standard ultrasound gel. The distance between the transducer and cells was less than 2 mm (Fig. 1B). The ultrasound intensity near the transducer surface has been proven to be constant throughout the cross sectional area, as reported previously [28]. The LIPUS treatment protocols are summarized in Fig. 1.

The parameters of LIPUS used in the present study consisted of a 1.5 MHz ultrasound signal with 200 µs burst sine wave, pulsed 1∶4 (2 ms ‘‘on’’ and 8 ms ‘‘off’’) with repetition rate at 1.0 kHz, and an intensity of 30, 60, and 90 mW/cm^2^ spatial and temporal average. The percentage permeability of ultrasound through the cell plate bottom in the presence of ultrasonic coupling gel was ∼63.56±2.02%, as determined previously using the ultrasound power meter (UPM-DT-1 AV; Ohmic Instruments, Easton, MD, USA) [29]. All experiments were carried out at 37°C. Sham controls were handled in the same way as the treated ones, but the ultrasound generator was not switched on.

### Optimization of LIPUS treatment dosage

HPDLCs growing on 6-well plates were assigned into 9 experimental groups and a non-treated control group, with 6 replicates for each group. Cells in the treatment groups were exposed to LIPUS at different intensities (i.e., 30, 60, or 90 mW/cm^2^) for 10, 20, or 30 min/day for 5 consecutive days. The control group was not treated with LIPUS. After treatment, the cells were harvested and tested for ALP activity as described below. The LIPUS treatment dosage that resulted in a maximum increase in the ALP activity was considered as an optimal treatment condition used in the following experiments.

### Measurement of ALP activity and osteocalcin production

Similarly, cells were cultured and treated in the presence and absence of daily LIPUS stimulation (90 mW/cm^2^ -20 min/d). At 3, 5, 7, 9, 11, and 13 days after LIPUS treatment, one plate from each group was randomly selected for measurement of ALP activity. The culture medium was collected and stored at -80°C until use. The cells were detached with trypsin, incubated in 0.2% Triton for 24 h at 4°C, and subjected to sonication (Shanghai Bilon Instruction Co., Ltd., Shanghai, China). ALP activities in the media and cell lysates were measured by determining the formation of p-nitrophenol from p-nitrophenol phosphate using a commercially available kit (Nanjing Jiancheng Bioengineering Institute, Nanjing, China) according to the manufacturer’s instructions. One unit of ALP activity was defined as the amount of enzyme required to produce 1.0 mmol of p-nitrophenol per minute.

Cells were cultured and treated in the presence and absence of daily LIPUS stimulation (90 mW/cm^2^ -20 min/d), for up to 15 days. The cell culture media were collected from each group at 5, 7, 9, 11, 13, and 15 days after LIPUS treatment (90 mW/cm^2^ -20 min/d). Osteocalcin levels in the media were measured using a commercially available radioimmunoassay kit (Beijing Atom High Tech Co., Ltd., Beijing, China) following the manufacturer’s instructions.

### Analysis of mRNA expression

Cells growing on 6-well plates were assigned into three groups instead of two groups as mentioned above. The LIPUS-treated group and non-treated control group were the same as those described previously, and cells in the third group were treated with both daily LIPUS stimulation (90 mW/cm^2^ -20 min/d) and 10 ug/mL blocking antibody against integrinβ1 (BD Biosciences, USA), a specific integrinβ1 inhibitor [30], [31]. 7 days later, total RNA was isolated from the cultured cells after LIPUS treatment (90 mW/cm^2^ -20 min/d) for 5 consecutive days, using Trizol reagent (Invitrogen, Carlsbad, CA, USA) according to the manufacturer’s instructions. cDNA was reverse transcripted from 1 µg total RNA samples. The specific primers for amplification of human Runx2 [32], integrinβ1 [33], and house gene (i.e. glyceraldehyde-3-phosphate dehydrogenase, GAPDH) cDNA are summarized in Table 1. The cycling conditions were as follows: initial denaturation at 95°C for 5 min, and then 29 cycles of denaturation at 94°C for 30 s, annealing at 50°C for 30 s, and elongation at 72°C for 1 min. Aliquots of PCR products were run on a 2% agarose gel containing ethidium bromide and visualized with ultraviolet light. Signals were densitometrically quantified and results are expressed relative to GAPDH mRNA levels. The experiments were performed in triplicate.

**Table 1:** Ostogenic differentiation-related gene expression: product size of Runx2 and Intergrin β1.

Genes	Primer sequences	Product size (bp)
GAPDH	Forward: 5′-tgaacgggaabctcactgg-3′	307
	Reverse: 5′-tccaccaccctgttgctgta-3′	
Runx2	Forward: 5′-ggcaagagtttcaccttgacc-3′	164
	Reverse: 5′-tcactgaggcggtcagagaac-3′	
Intergrin β1	Forward: 5′-agtgaacagaactgcaccagc-3′	181
	Reverse: 5′-tcctccagccaatcagtgatc-3′	

### Assessment of the effects of integrinβ1 inhibitor on LIPUS-induced ALP activity, osteocalcin production

HPDLCs growing on 6-well plates were assigned into three groups as mentioned above, cultured for up to 14 days. Medium was changed every 2 or 3 days, and at Days 8, 11, and 14, the culture medium of each group was collected and stored at -80°C until use. ALP activities in cell lysates were measured at 7 and 14 days. Total osteocalcin production in the stored media were measured.

### Alizarin red staining of calcified nodules

The ability of cells to produce mineralised matrix and calcium nodules is a key factor for osteogenic differentiation and bone regeneration [34]. Whether mineralisation of matrix and nodules occurred was determined using alizarin red staining. Alizarin red is a common histochemical technique used to detect calcium deposits in mineralised tissues and cultures [35]. Cells were placed into 6-well plates containing DMEM supplemented with 10% FBS, 100 µg/mL streptomycin, 100 U/mL penicillin, 50 ug/ml ascorbic acid (Sigma, USA) and 10 mM β-glycerophosphate (Sigma, USA), at a density of 5.0×10^5^ cells/cm^2^, to induce mineralized matrix deposition. And the experiment was assigned into three groups and treated similarly as mentioned above. An Alizarin Red S staining kit (GENMED, Shanghai, China) was used to analyse mineralized matrix deposition on the cells after 21 d of culture following the manufacturer’s instructions. Images of the stained cells were observed and captured using an inverted microscope. The formation of one calcium nodule with diameter greater than 1 mm was considered as one counting unit [36].

### Statistical analysis

All data are presented as the mean±standard deviation (SD). Significant differences between experimental group and controlled group were determined by the Student's *t*-test. Differences in ALP activities among the LIPUS-treated groups with different ultrasonic intensities and daily treatment durations were assessed by two-way analysis of variance (ANOVA) followed by the Student-Newman-Keuls post hoc test. A value of *P* <0.05 was considered statistically significant.

## Results

### Morphology and characterization of hPDLCs

**Figure 2:**
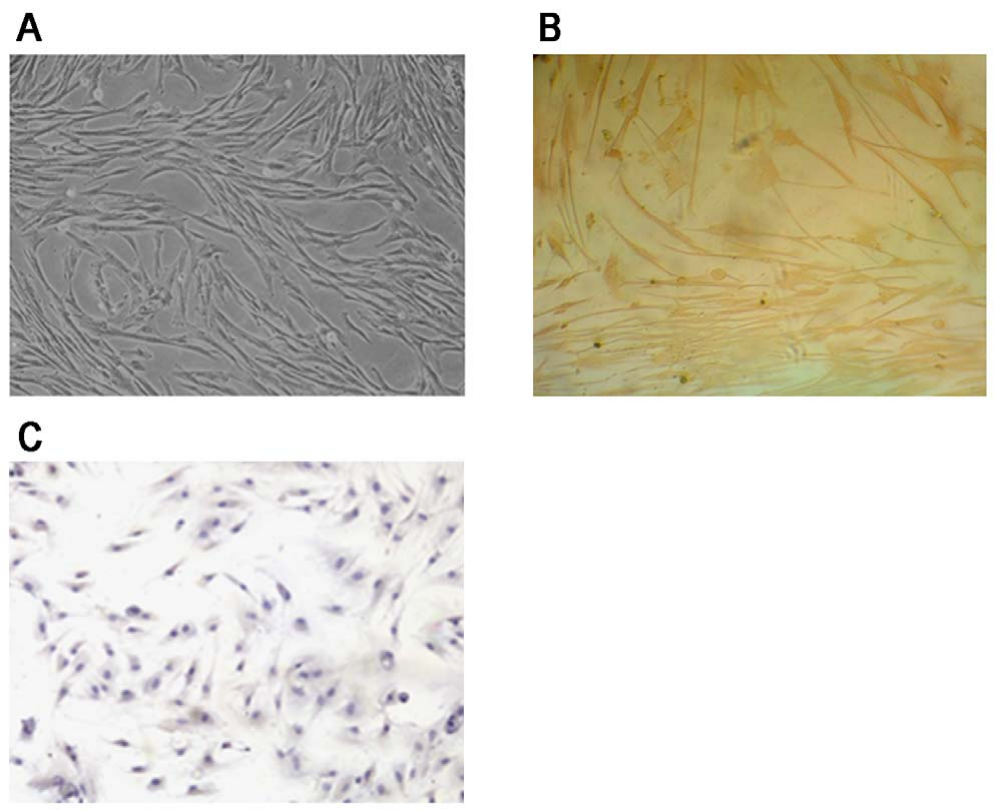
HPDLCs were characterized morphologically under an inverted microscope and examined immunohistochemically using anti-vimentin antibody and anti-keratin antibody. (A) HPDLCs were long-shuttle in shape after generation (×100). (B) Positive immunohistochmical staining for Vimentin on hPDLCs (×100). (C) Negtive immunohistochmical staining for Keratin on hPDLCs (×100).

PDLCs were long-shuttle shaped fibroblast-like cells. HPDLCs were long-shuttle in shape after generation, simultaneously few hPDLCs were polygon in shape(Fig. 2A). Anti-vimentin protein antibody was positive (Fig. 2B) and anti-keratin antibody of hPDLCs was negative (Fig. 2C). These approved that the cells were fibroblasts of connective tissue derived from mesoblast.

### Optimal daily dose for LIPUS treatment

**Figure 3:**
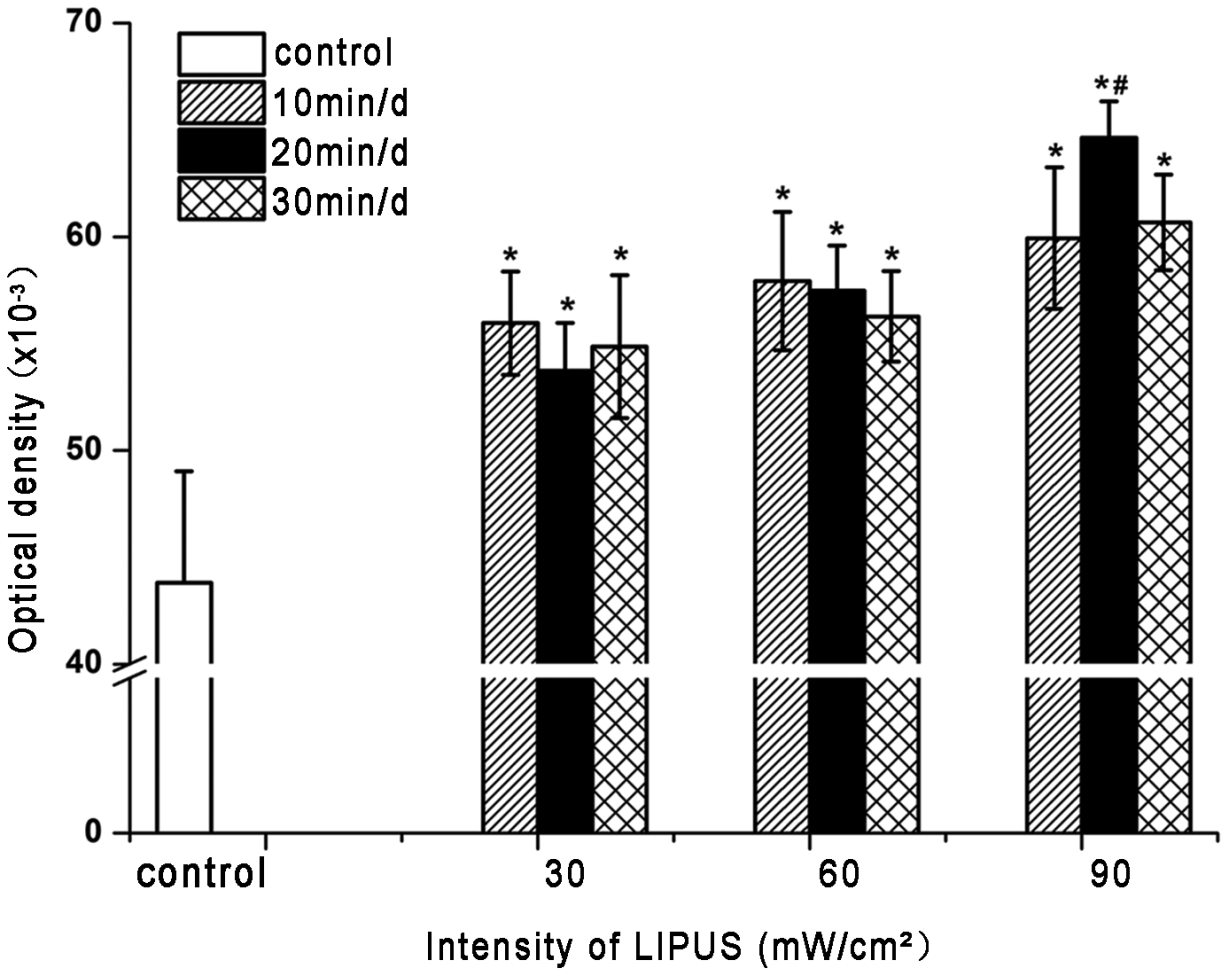
Effect of different daily dosages of LIPUS on ALP activities of hPDLCs. HPDLCs were treated with LIPUS at an intensity of 30, 60, or 90/cm^2^ for 10, 20, or 30 min/day for 5 consecutive days. ALP activity was determined by colorimetric assay using a commercially available ALP kit. LIPUS treatment caused a significant increase in the ALP activity, as compared to non-treated control group and LIPUS at the intensity of 90 mW/cm^2^ with 20 min/day was more effective. The data are presented as the mean±SD of three separate experiments. ^*^ *P*<0.05 vs. the control group; ^#^ *P*<0.05 vs. any of the other LIPUS-treated group.

LIPUS treatment caused a significant (*P*<0.05) increase in the ALP activity of hPDLCs, as compared to non-treated control cells (Fig. 3). Moreover, the ALP activity of hPDLCs markedly changed with both intensity and exposure time of LIPUS. The Student-Newman-Keuls test showed that hPDLCs exposed to LIPUS at an intensity of 90 mW/cm^2^ for 20 min per day had a significantly (*P*<0.05) greater increment in ALP activity, compared with any of the other LIPUS-treated groups (Fig. 3). Therefore, the LIPUS intensity of 90 mW/cm^2^ and the treatment duration of 20 min/day were applied in the following experiments.

### Dynamic changes of ALP activity and osteocalcin production

**Figure 4:**
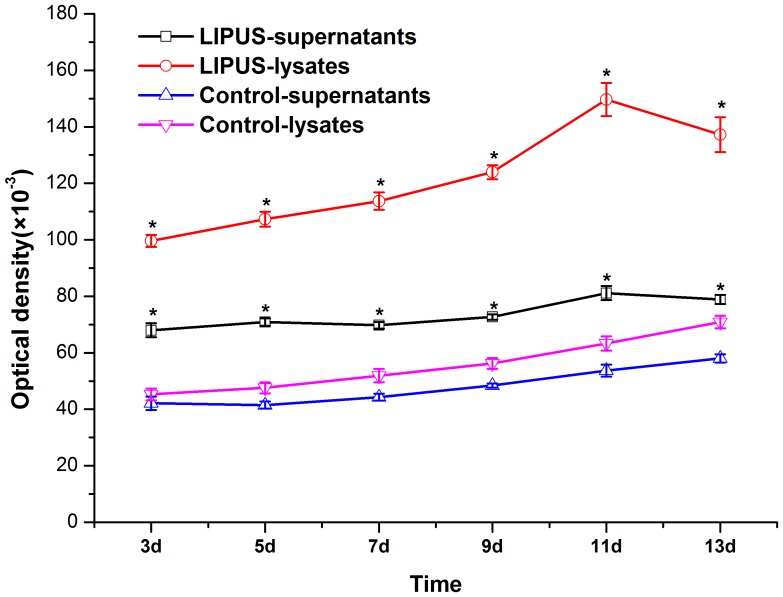
Time course of ALP activity in hPDLCs treated with LIPUS for 13 days. The cells were treated with or without LIPUS and ALP activities in the cultured cells and supernatants were determined at 3, 5, 7, 9, 11, and 13 days of culture. The ALP activities in lysates and supernatants of LIPUS-treated cells started to increase at days 3 and 7, respectively, and peaked at day 11, and the ALP activity was significantly higher in the LIPUS stimulation group than that in the control group at each time point. The data are presented as the mean±SD of three separate experiments. ^*^ *P*<0.05 vs. the control group.

ALP activity in hPDLCs was examined up to 13 days of culture with and without LIPUS stimulation. As shown in Fig. 4, the ALP activities in both lysates and supernatants from cells without LIPUS treatment increased slightly after the 13-day culture. In the LIPUS-treated groups, the ALP activities in lysates and supernatants started to increase at days 3 and 7, respectively, and simultaneously peaked at day 11. Of note, cells with LIPUS treatment had significantly (*P*<0.05) higher ALP activities than those without at each time point (Fig. 4).

**Figure 5:**
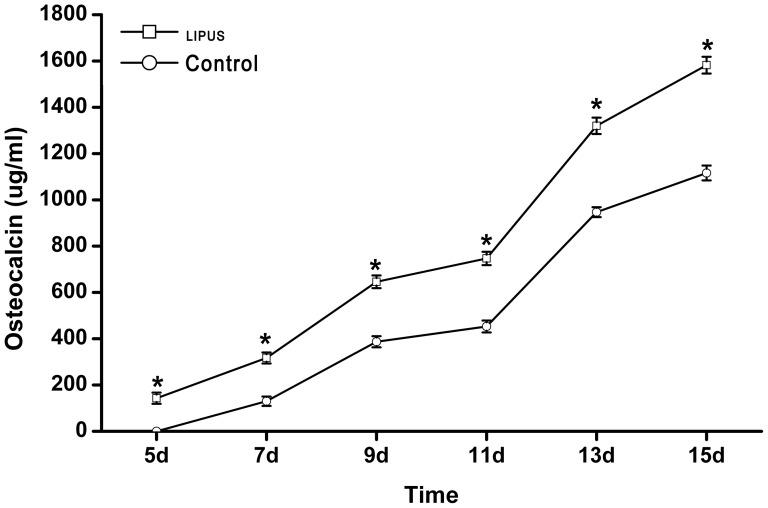
Time course of osteocalcin production from hPDLCs exposed to LIPUS. The cells were treated with or without LIPUS and osteocalcin levels in the conditioned media were determined by radioimmunoassay at 5, 7, 9, 11, 13, and 15 days of culture. The release of osteocalcin into the media was continuously increased from day 5 and the production of osteocalcin was significantly higher in the LIPUS stimulation group than that in the control group at each time point. The data are presented as the mean±SD of three separate experiments. ^*^ *P*<0.05 vs. the control group.

Fig. 5 shows the time course of the osteocalcin production of hPDLCs treated with or without LIPUS stimulation. In both the LIPUS and control groups, the release of osteocalcin into the media was continuously increased from day 5 to the end of the culture experiment. Notably, LIPUS treatment caused a significantly (*P*<0.05) greater production of osteocalcin at each time point after day 5, as compared to non-treated cells (Fig. 5).

### Effects of LIPUS on Runx2 and integrin β1 mRNA expression

**Figure 6:**
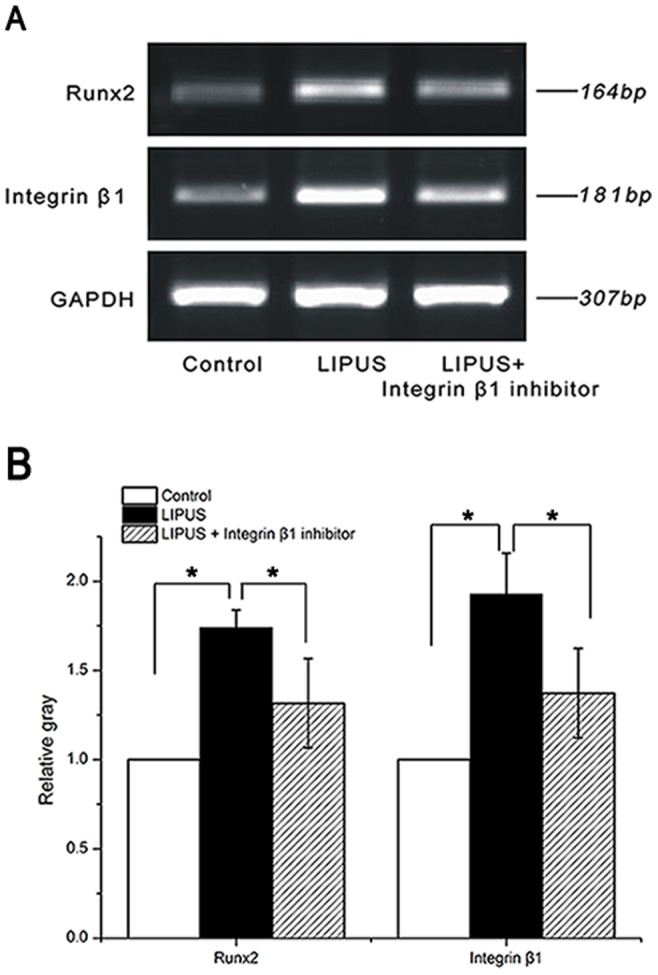
Effects of LIPUS on Runx2 and integrin β1 mRNA expression. HPDLCs were assigned into LIPUS-treated group, non-treated control group and the combination group treated wtih LIPUS and integrinβ1 inhibitor, cultured for up to 7 days. The abundance of Runx2 and integrin β1 transcripts was determined by semi-quantitative RT-PCR assay. (A) Representative agarose gels showing gene expression changes between the three groups. (B) Densitometric analysis of gel images normalized against the GAPDH gene. LIPUS caused a significant increase in the mRNA expression of Runx2 and integrin β1, while a significant decline when the integrinβ1 inhibitor was used. The data are presented as the mean±SD of three separate experiments. ^*^ *P*<0.05 vs. any of the other two groups.

The effects of LIPUS stimulation on the mRNA expression of Runx2 and integrin β1 were assessed during a 7-day culture period. Semi-quantitative RT-PCR analysis revealed that LIPUS-treated hPDLCs expressed significantly (*P*<0.05) higher levels of Runx2 and integrin β1 mRNA, compared with any of the other two groups (Fig. 6A, B).

### Effects of integrinβ1 inhibitor on LIPUS-induced ALP activity, osteocalcin production as well as calcium deposition

**Figure 7:**
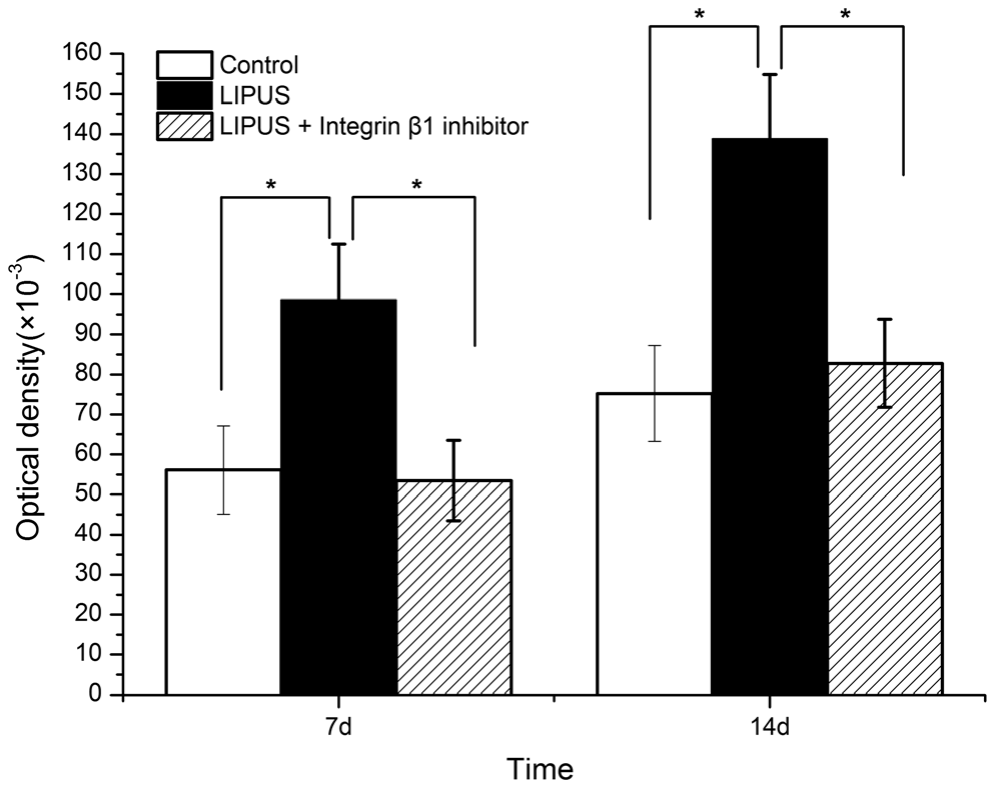
Effects of integrinβ1 inhibitor on LIPUS-induced ALP activity. ALP activities in cell lysates were measured at 7 and 14 days. ALP activity was significantly higher in the LIPUS-treated cells than that in the control group and the combination group treated wtih both LIPUS and integrinβ1 inhibitor. The data are presented as the mean±SD of three separate experiments. ^*^ *P*<0.05 vs. any of the other two groups.

At Days 7, 14 of culture, ALP activities in cell lysates was measured. Regardless of integrinβ1 inhibitor, ALP activity was significantly (*P*<0.05) higher in the LIPUS-treated cells than that in the non-treated control cells (Fig. 7). And the ALP activity of cells treated with both daily LIPUS stimulation and blocking antibody against integrinβ1 was less than that in the LIPUS-treated group (*P*<0.05).

**Figure 8:**
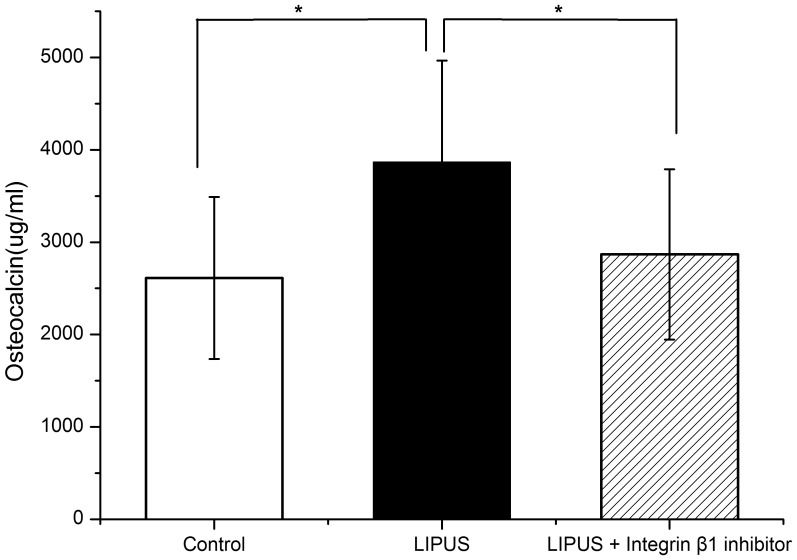
Effects of integrinβ1 inhibitor on LIPUS-induced osteocalcin production. Total osteocalcin production in the stored culture media were measured. LIPUS stimulation promoted the production of osteocalcin, while a significant decline when the integrinβ1 inhibitor was used. The data are presented as the mean±SD of three separate experiments. ^*^ *P*<0.05 vs. any of the other two groups.

At Days 8, 11, and 14, the culture medium of each group was collected and total osteocalcin production in the media of each group were measured. The osteocalcin content in the media of cells just exposed to LIPUS was the highest (*P*<0.05), compared with any of the other two groups (Fig. 8).

**Figure 9:**
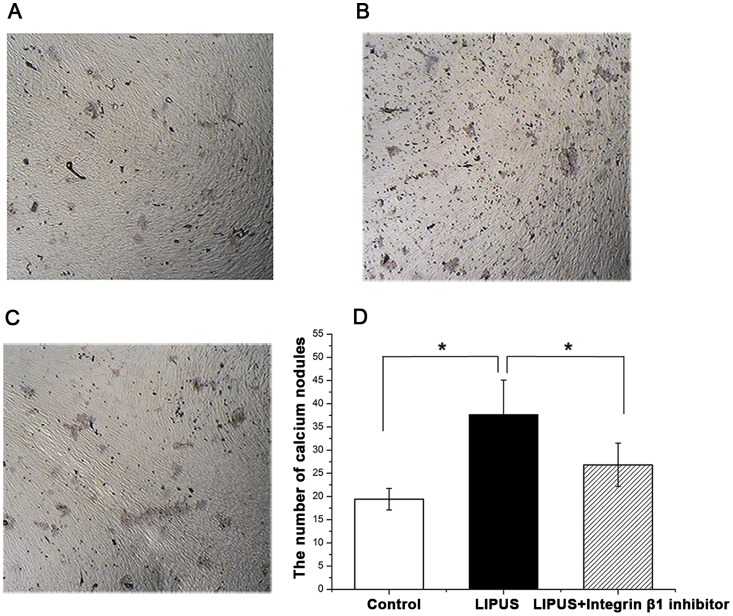
Detection of calcium nodules by Alizarin red staining in hPDLCs. HPDLCs were assigned into LIPUS-treated group, non-treated control group and the combination group treated wtih LIPUS and integrinβ1 inhibitor, cultured with the osteoblast inducing conditional media for up to 21 days. The mineralized matrix deposition was determined using alizarin red staining. (A) The formation of calcium nodules in non-treated control cells. (B) The formation of calcium nodules in LIPUS-treated cells. (C) The formation of calcium nodules in cells treated wtih both LIPUS and integrinβ1 inhibitor. (D) LIPUS stimulation promoted formation of calcium deposition in hPDLCs, while a significant decline when the specific integrinβ1 inhibitor was used. The data are presented as the mean±SD of three separate experiments. ^*^ *P*<0.05 vs. any of the other two groups.

Mineralized nodule formation was determined at day 21 of culture. Many red calcium nodules in various size were found in the cells of all three groups (Fig. 9A, B, C). The staining of mineralized nodules by alizarin red was clearly (*P*<0.05) higher in the cells stimulated with LIPUS only, compared with any of the other two groups (Fig. 9).

## Discussion

LIPUS is a form of mechanical energy that is transmitted through and into living tissues as an acoustic pressure wave, resulting in biochemical events at the cellular level, and has been widely accepted as an efficient and safe therapeutic approach in bone regeneration [17]. In contrast to high-intensity continuous ultrasound, LIPUS exerts no or minimal adverse cell [37] or tissue effects [38]. Although the absorption of the ultrasound signal may result in energy conversion to heat, this effect is extremely small for low intensity pulsed ultrasonic waves [37], [38], [39]. Several previous studies reported that the acceleration of bone regeneration *in vivo* and the osteoinductive response *in vitro* induced by LIPUS were through its non-thermal effects [40], [41]. Furthermore, Chang *et al.* carried out a comparative study of the effects of LIPUS and microwave hyperthermia stimulation on osteogenesis, and their results demonstrated that ultrasound stimulation-induced new bone formation was not mediated via hyperthermia [42]. In the present study, the percentage permeability of ultrasound through the bottom of the 6-well culture plate was approximately 63±2%. In other words, if a power density of 90 mW/cm^2^ was applied, the actual acoustic power acting on cultured cells was approximately 57.2 mW/cm^2^, suggesting that the ultrasonic intensity used in this study was low. In previous studies, LIPUS with various intensities has been reported and the majority of the in vitro studies used was 30 mW/cm^2^ [28], but our study, which made a selection of daily LIPUS treatment dosage before the formal experiment, showed that LIPUS at the intensity of 90 mW/cm^2^ with 20 min/day was more effective.

ALP is a hydrolase enzyme which can hydrolyze the ester bond of organic phosphate compound under alkaline conditions and plays an important role in the calcification of bone. The enzyme not only hydrolyzes substances that inhibit calcification, such as pyrophosphate and ATP, but it is also indispensable for producing the increased phosphate concentration required for hydroxyapatite crystallization [43]. In addition, high ALP activity correlates with matrix formation in osteoblasts prior to the initiation of mineralization, as described by Gerstenfeld et al [44]. ALP activity is generally used as an early-stage marker of osteogenic differentiation [45]. It has been documented that the ALP activity in osteoblasts or osteoblast-like cells began to increase from day 5 after daily transient LIPUS stimulation, indicative of enhanced osteogenesis [21], [37]. Our results consistently showed that there was a significant increase in ALP activity in LIPUS-treated hPDLCs at each time point studied, compared with non-treated cells. As ALP activities in lysates and supernatants represent the synthesis and secretion levels of ALP, respectively, our results indicated that LIPUS-treated PDLCs had enhanced synthesis and secretion of ALP at days 3 and 7, respectively. Moreover, both ALP synthesis and secretion peaked at day 11, followed by a slight decline until day 13, when late-stage osteogenic differentiation of PDLCs may occur. In addition to ALP activity, we found that LIPUS treatment caused a significant and time-dependent elevation in the release of osteocalcin from PDLCs, as compared to non-treated cells. Osteocalcin, also known as bone gamma-carboxyglutamic acid-containing protein, is a noncollagenous protein found in bone and dentin. As osteocalcin is secreted solely by osteoblasts and thought to play a role in bone-building and bone mineralization [46], it is often used as a late-stage marker of bone formation process [47] or osteogenic differentiation [45]. In agreement with our findings, several previous studies have reported an acceleration of osteocalcin expression in osteoblasts [48] and odontoblast-like cells [49] exposed to ultrasound. Taken together, we provide evidence that daily transient LIPUS stimulation can enhance osteogenic differentiation of hPDLCs.

To better understand the osteogenic activity of LIPUS, we investigated the expression changes of osteogenesis-related genes after LIPUS treatment. Runx2, a Runt domain transcription factor family member, is an essential transcription factor in the osteogenic pathway [50]. Runx2 is capable of transcriptionally activating multiple osteoblast-specific genes, including ALP, osteocalcin, collagen I, osteopontin, and bone sialoprotein [51]. Sant'Anna *et al.* reported that rat bone marrow stromal cells exposed to LIPUS had a significant increase in Runx2 expression, compared with non-treated cells [32]. In line with this study, we also observed that the mRNA expression level of Runx2 was significantly raised upon LIPUS stimulation. Runx2 is a key target of mechanical stimulation in hPDLCs [52], contributing to bone remodeling. Li *et al.* demonstrated that cyclic tensile stress enhanced osteogenic differentiation of hPDLCs via activation of ERK1/2-Elk1 MAPK pathway and upregulation of Runx2 expression [53]. These studies, combined with our findings, suggest that LIPUS-induced osteogenic differentiation of hPDLCs is associated with the upregulation of Runx2. However, it remains to be further clarified to what extent Runx2 contributes to such osteogenic differentiation of hPDLCs.

The mechanisms involved in LIPUS-stimulated tissue repair have not been elucidated yet; however, it is recognized that the anabolic biophysical effects caused by LIPUS are most likely to be caused by mechanical stress and/or fluid micro-streaming impacting on the cellular plasma membrane, focal adhesion and cytoskeletal structures to trigger intracellular signal transduction and subsequent gene transcription [17], [54]. To further elucidate the underlying mechanisms through which hPDLCs sense and convert LIPUS stimulation into cellular responses, we investigated the gene expression changes of integrin β1 after LIPUS treatment. Integrins, which connect the cytoskeleton to the extracellular matrix and mediate a variety of signaling cascades, are involved in the transduction of mechanical stimuli to biochemical signals [55]. A number of integrin subunits, specifically integrin β1 are abundantly expressed in osteoblasts or osteoblast-like cells [56], and hPDLCs [57]. Integrin β1 is implicated in the remodeling of periodontium in response to mechanical stimulation [58]. Takeuchi *et al.* [59] showed that LIPUS stimulated the growth of chondrocytes via integrin β1 that acted as a mechanoreceptor on the cell membrane. Our present data showed that LIPUS stimulation resulted in a significant increase in the mRNA expression of integrin β1 in hPDLCs, while a significant decline when the function-blocking antibody directed against integrinβ1 was used. Similarly, the mRNA expression level of integrin β1 was significantly augmented in human mesenchymal stem cells exposed to LIPUS [33]. Furthermore, in this study, the function-blocking antibody directed against integrinβ1 was utilized to study the effects of integrinβ1 inhibitor on LIPUS-induced ALP activity, osteocalcin production as well as calcium deposition. And the result suggested that blockade of integrinβ1 significantly attenuated LIPUS-induced Runx2 expression, ALP secretion, osteocalcin production and calcium deposition.

These findings collectively suggest that activation of integrin β1-dependent signaling transduction may be an important mechanism for the osteogenic activity of LIPUS in hPDLCs. A study performed by our group demonstrated that the p38 MAPK pathway, an important mediator of bone differentiation, was involved in the process of LIPUS-induced osteogenic differentiation of hPDLCs [15]. Thus, we hypothesized that when ultrasound is transmitted to integrin molecules, the mitogen-activated protein kinase(MAPK) pathway, one of the principle signal transduction cascades to be associated with mechanotransduction[60], [61], might be in turn activated and then osteogenetic differentiation of hPDLCs began a process to initiate, without exclusion of other regulating pathways. Nevertheless, the signaling pathways are multiple, and more evidences for the plausible role of integrin β1 signaling in LIPUS-induced hPDLC osteogenesis need to be obtained.

In conclusion, our data demonstrates that LIPUS stimulation facilitates osteogenic differentiation of hPDLCs, associated with activation of integrin β1-dependent signaling transduction and upregulation of Runx2 expression. These results highlight the therapeutic implications of ultrasound stimulation in periodontal regeneration. However, further studies are needed to elucidate the biological roles of integrin β1 signaling in LIPUS-induced osteogenic differentiation of hPDLCs.
